# Causal Association Between Monounsaturated Fatty Acids and Lung Cancer: A Two‐Sample Mendelian Randomization Study

**DOI:** 10.1111/crj.70038

**Published:** 2024-12-10

**Authors:** Shaofeng Zhang, Jia Jiang, Xiping Wu, Jiayi Liu, Wei Lei, Siqin Chen, Yaling Zeng, Xiang Liu, Qiang Xiao

**Affiliations:** ^1^ Pulmonary and Critical Care Medicine Shunde Hospital, Southern Medical University (The First People's Hospital of Shunde, Foshan) Foshan Guangdong China; ^2^ Pulmonary and Critical Care Medicine, Zhujiang Hospital Southern Medical University Guangzhou Guangdong China; ^3^ Departments of Hematology Shunde Hospital, Southern Medical University (The First People's Hospital of Shunde, Foshan) Foshan Guangdong China

**Keywords:** causality, lung cancer, Mendelian randomization, monounsaturated fatty acids

## Abstract

**Objective:**

This study aims to investigate the potential causal relationship between monounsaturated fatty acids (MUFAs) and lung cancer.

**Methods:**

Genetic data on MUFAs and pathological subtypes of lung cancer were extracted from genome‐wide association studies (GWAS). The primary analysis utilized inverse‐variance weighted analysis (IVW), with additional methods including the weighted median method, MR‐Egger regression method, and weighted model method. Sensitivity analysis was conducted to assess the robustness of the findings.

**Results:**

The inverse variance–weighted (IVW) analysis of monounsaturated fatty acids in relation to lung adenocarcinoma yielded an odds ratio (OR) of 1.059 with a 95% confidence interval of 0.960 to 1.168 and a *p* value of 0.252. Similarly, for lung squamous cell carcinoma, the IVW analysis produced odd ratios of 0.884, 95% confidence intervals of 0.747 to 1.045, and a *p* value of 0.148. In the case of small cell lung cancer, the odds ratio was 0.936, the 95% confidence interval was 0.751 to 1.166, and the *p* value was 0.554.

**Conclusion:**

It can be concluded that there is no direct causal relationship between monounsaturated fatty acids and the development of lung cancer.

## Graphical abstract

The findings from the Mendelian randomization analysis indicate that there is no direct causal association between monounsaturated fatty acids (MUFAs) and lung cancer. Nevertheless, it is possible that MUFAs may be influenced by iron levels or modulate immune responses, thereby indirectly impacting the progression of lung cancer.

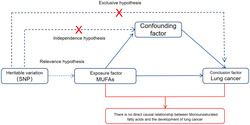

## 1 Introduction

Lung cancer is a prevalent malignant neoplasm worldwide, ranking second in global incidence rates of malignancies and serving as a leading cause of cancer‐related mortality [1, 2]. The escalating trend in diagnosis rates underscores the significance of lung cancer as a pressing public health concern in China, where its increasing morbidity and mortality rates impose a substantial burden on society [3]. Based on various pathological classifications, lung cancer can be categorized into non–small cell lung cancer, which accounts for 85% of total diagnoses, and small cell lung cancer, which accounts for 15% of total diagnoses. Among non–small cell lung cancer cases, adenocarcinoma is the most prevalent type, followed by squamous cell carcinoma [4]. Given the significant prevalence and mortality rates associated with lung cancer, it is crucial to conduct screenings for unidentified risk factors, such as dietary habits.

Monounsaturated fatty acids (MUFAs) are a type of fatty acid characterized by the presence of a double bond in their chemical structure, with prominent examples including oleic acid (OA) and palmitoleic acid (PA). These fatty acids are commonly found in various dietary sources. Research has demonstrated that single unsaturated fatty acids can potentially contribute to the prevention and treatment of type 2 diabetes and reduction of cardiovascular disease [5, 6]. However, their susceptibility to oxidation in the body, leading to the generation of superoxide, poses a potential safety hazard and risk of adverse reactions. Palmitoleic acid, for instance, can enhance insulin sensitivity and reduce fat accumulation, showing anti‐inflammatory effects. MUFAs are considered beneficial for cardiovascular health and may lower the risk of atherosclerosis [7].

In studies examining the relationship between monounsaturated fatty acids and cancer, it was observed that olive oil, which is rich in oleic acid, plays a modulating role in cancer development [8]. The exogenous introduction of monounsaturated fatty acids or the upregulation of stearoyl‐CoA desaturase 1 (SCD1) results in an increase in endogenous monounsaturated fatty acids, thereby enhancing the ability of ovarian cancer cells to withstand ferroptosis and ultimately promoting cancer progression [9]. Polyunsaturated fatty acids (PUFAs) are characterized by the presence of two or more double bonds. Research indicates a potential link between a high consumption of polyunsaturated fatty acids and a decreased risk of lung cancer, whereas the association between MUFAs and lung cancer risk remains inconclusive [10]. Furthermore, observational studies may be influenced by various confounding variables, and the direct causal relationship between monounsaturated fatty acids and lung cancer risk requires further investigation.

The Mendelian randomization study (MR) utilizes instrumental variables, specifically the genetic variation of single nucleotide polymorphisms (SNPs), to investigate factors that influence outcome variables. By employing SNPs as genetic factors, this approach mitigates biases stemming from environmental, investigator, behavioral, and social factors typically present in observational studies. Furthermore, Mendelian research provides a clear and unidirectional assessment of causality, less susceptible to the confounding effects of reverse causation.

Previous research has indicated a potential correlation between lung cancer and monounsaturated fatty acids, although these findings may be influenced by confounding variables or the potential for reverse causation. This study employs Mendelian randomization methods to investigate the direct causal relationship between three pathological types of lung cancer, monounsaturated fatty acids, and lung cancer.

## 2 Materials and Methods

### 2.1 Selection of Genetic Variants

This study utilized MUFAs as the exposures of interest and identified significant MUFAs SNPs as instrumental variables. The findings indicated a relationship with lung cancer through Mendelian randomization analysis. Heterogeneity was assessed using Cochran's *Q* test, and sensitivity analysis was conducted using the MR‐Egger intercept and one method.

**FIGURE 1:**
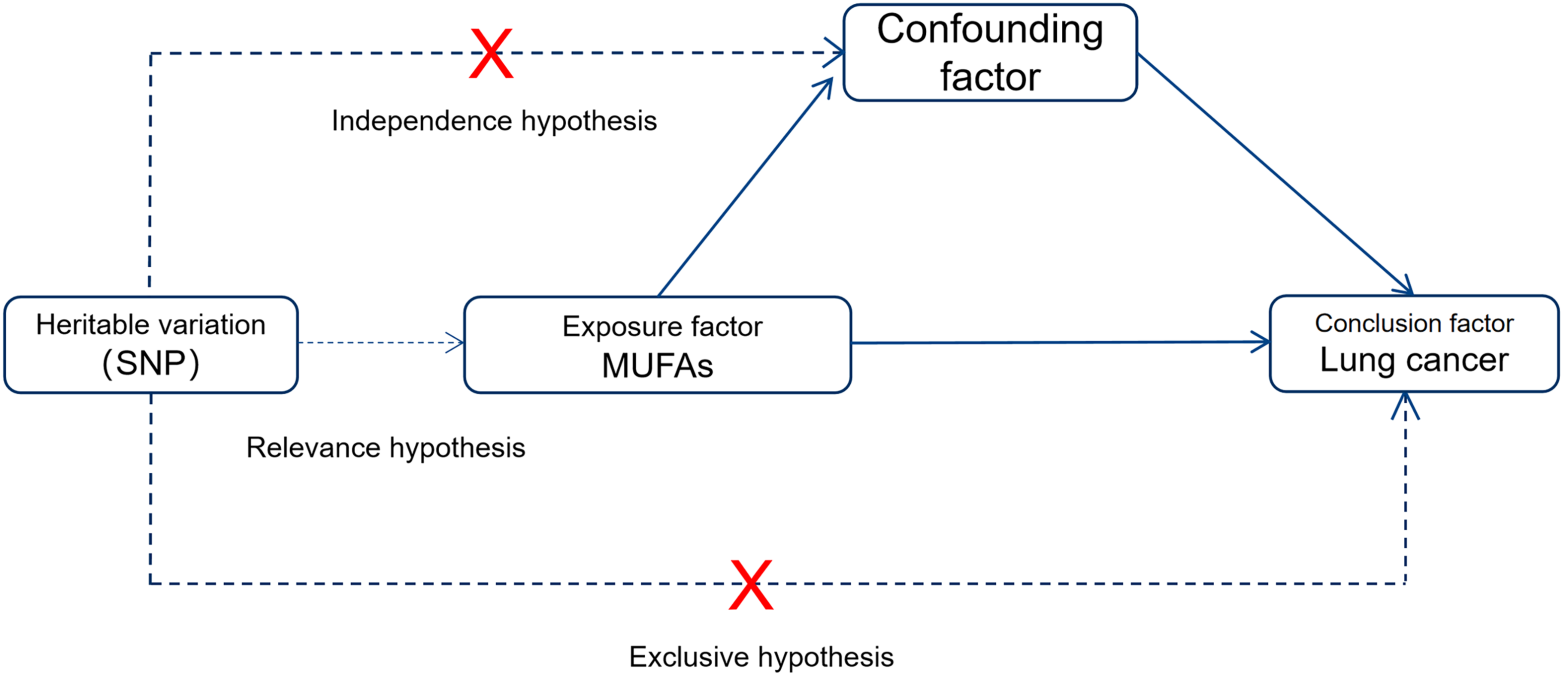
Study design.

The study design depicted in Figure 1 is grounded in the fundamental principles of Mendelian randomization methods and must adhere to the following three hypotheses [11]: (1) The instrumental variable must have a direct relationship with the exposure factors; (2) the instrumental variable should not be influenced by external confounding factors; (3) the instrumental variable must solely impact outcome factors through exposure factors.

### 2.2 Data Sources

MUFAs and lung cancer data were obtained from the GWAS database (https://gwas.mrcieu.ac.uk/), utilizing data sourced from publicly available research papers [12]. The original ethical approval documentation for this study has been acquired, and no further ethical review is deemed necessary. Specific data details can be found in Table 1. The analysis of over 110 000 samples provided data on MUFAs, whereas information on lung cancer was obtained through genotyping of 14 803 European patients with lung cancer and 12 262 controls, as well as genome‐wide association studies (GWAS) involving 29 266 patients and 56 450 controls across three distinct pathological types of lung cancer.

**TABLE 1:** Data sources and information.

GWAS ID	Year	Name	Population	Sample size	Number of SNPs
met‐d‐MUFA	2020	Monounsaturated fatty acids	European	114 999	12 321 875
ebi‐a‐GCST004744	2017	Lung adenocarcinoma	European	66 756	7 849 324
ebi‐a‐GCST004750	2017	Squamous cell lung carcinoma	European	63 053	7 838 805
ebi‐a‐GCST004746	2017	Small cell lung carcinoma	European	24 108	7 620 430

Abbreviations: GWAS, genome‐wide association studies; SNP, single nucleotide polymorphism.

### 2.3 Study Methods

#### 2.3.1 Screening of Instrumental Variables

The instrumental variables selected must adhere to the criterion of being associated with exposure factors, with a screening threshold set at *p* < 5 × 10^−8^. Additionally, these SNPs must satisfy linkage disequilibrium (LD) conditions of *r* ^2^ < 0.001 and kb > 10 000 to ensure their validity. Following screening, SNPs directly linked to outcome factors were excluded, whereas those associated with outcome factors were retained. Allele data for both exposure and outcome factors were harmonized, and palindromic SNPs were eliminated. The remaining SNPs were utilized in the subsequent Mendelian randomization analysis.

### 2.4 MR Analysis

The “Two Sample MR” package within R software version 4.3.1 was utilized for conducting MR analysis, primarily employing the inverse variance weighting method, along with supplementary methods such as MR‐Egger regression, weighted median, and weighted mode. Heterogeneity was assessed using the Cochran *Q* test, with the inverse variance weighted random effects model applied for *P* ≥ 0.05, and the inverse variance weighted fixed effects model utilized for *p* < 0.05.

### 2.5 Sensitivity Analysis

The MR‐Egger regression method was employed to assess pleiotropy. An MR‐Egger regression intercept with a *p* value greater than 0.05 suggests the absence of horizontal pleiotropy. The leave‐one‐out test was utilized to evaluate the impact of individual SNPs on outcome variables. In cases where a SNP demonstrated a significant effect, it was removed from the analysis and the MR analysis was repeated.

## 3 Results

### 3.1 Causal Relationship Between MUFAs and Lung Adenocarcinoma

Instrumental variables: According to the principle of “Screening of instrumental Variables” in this paper, 51 SNPs were finally included for subsequent Mendelian randomization analysis.

**FIGURE 2:**
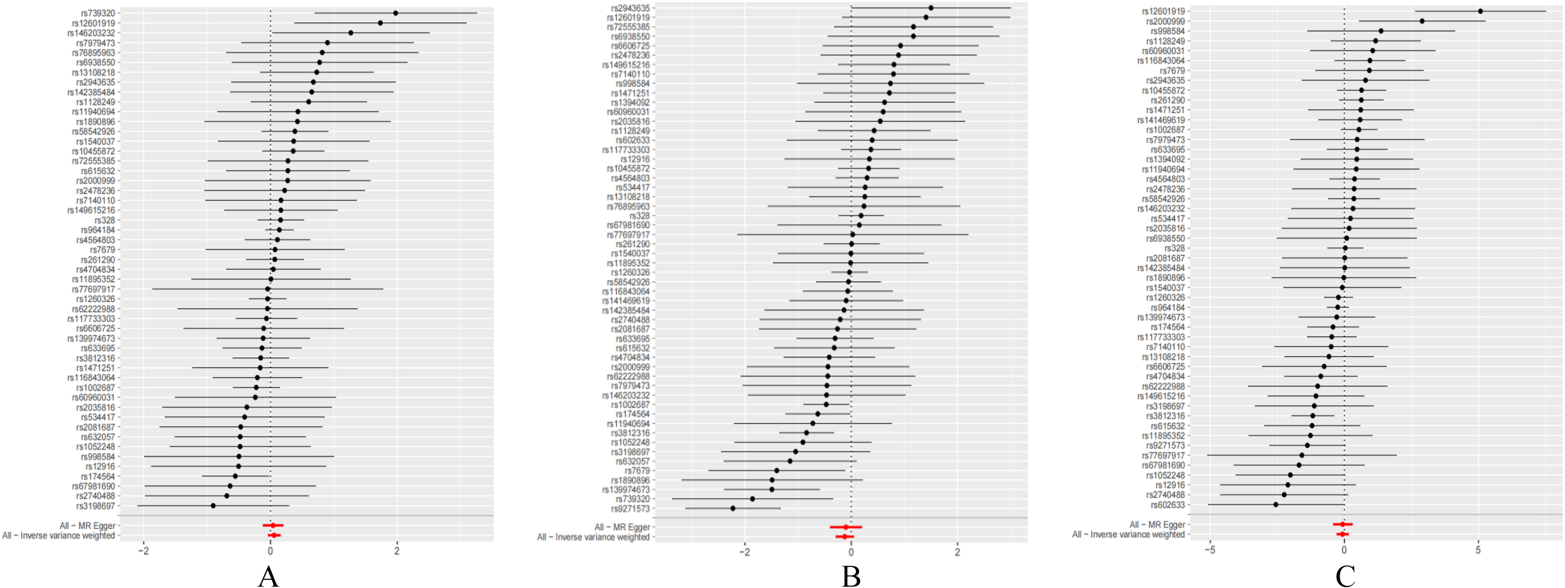
Three types of lung cancer pathology Mendelian randomization forest graph of analytical results. (A) Forest plot of causal effects of SNPS associated with MUFAs on lung adenocarcinoma. The significance of the red line is the MR Results of the MR‐Egger test and the IVW method. (B) Forest plot of causal effects of SNPS associated with MUFAs on lung squamous cell carcinomas. The significance of the red line is the MR Results of the MR‐Egger test and the IVW method. (C) Forest plot of causal effects of SNPS associated with MUFAs on small cell lung cancer. The significance of the red line is the MR results of the MR‐Egger test and the IVW method.

**FIGURE 3:**
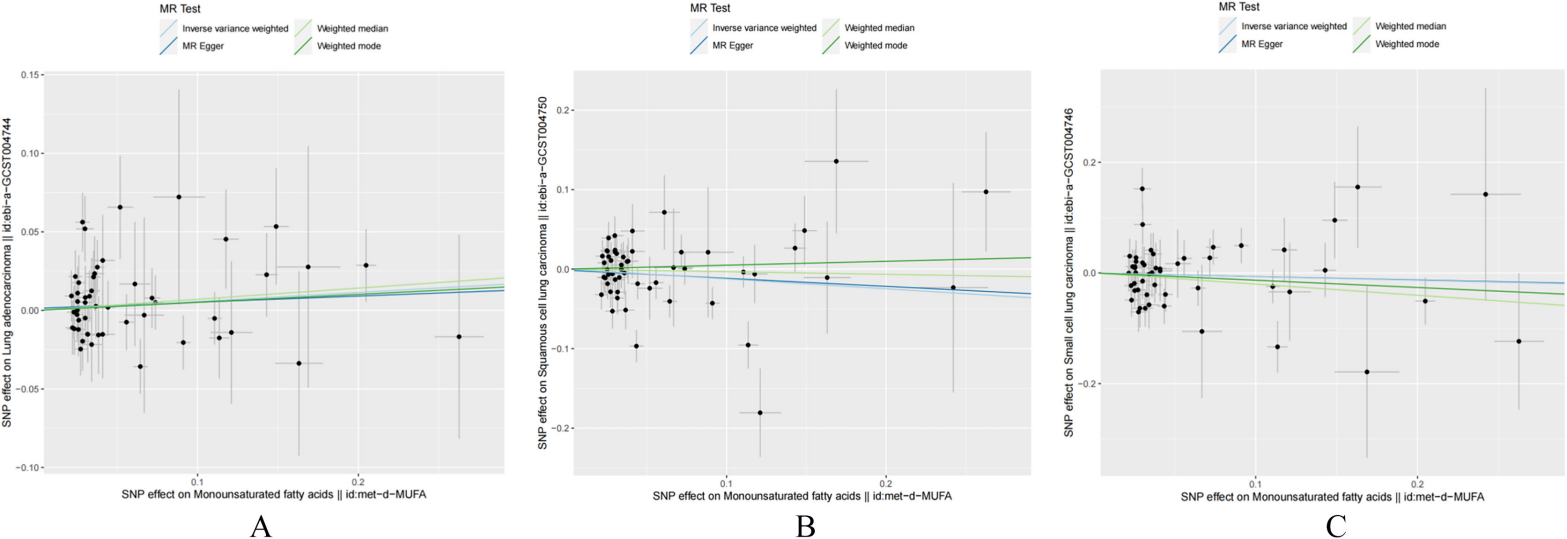
Scatter plot of the results of the Mendelian randomization analysis. (A) Causal association of MUFAs on lung adenocarcinoma. (B) Causal role of MUFAs on lung squamous cell carcinoma. (C) Causal effect of MUFAs on small cell lung cancer.

**FIGURE 4:**
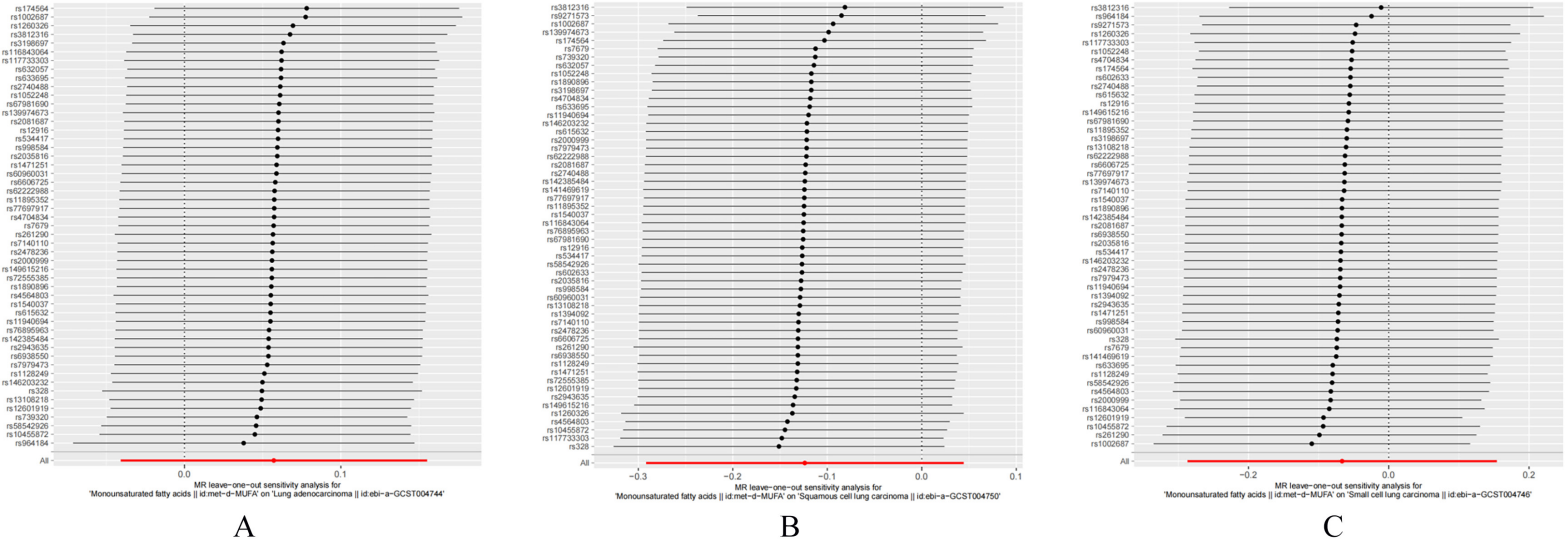
Leave‐one‐out method for sensitivity analysis. (A) Sensitivity analysis of MUFAs for lung adenocarcinoma. (B) MUFAs sensitivity analysis for lung squamous cell carcinoma. (C) MUFAs sensitivity analysis for small cell lung cancer.

The MR analysis and sensitivity analysis utilized the Cochran *Q* test, which yielded a *p* value of 0.3844. The random effect model using the inverse variance weighting method was selected with a *p* value of 0.252. Additionally, the MR‐Egger regression method, weighted median method, and weighted pattern method all produced results consistent with the inverse variance weighting method, with all *p* values exceeding 0.05. The results of the inverse variance–weighted (IVW) analysis yielded an odds ratio (OR) of 1.059 with a 95% confidence interval (CI) of 0.960 to 1.168, as depicted in Figure 2A, Figure 3A, and Table 2A. The MR Egger regression intercept *p* value of 0.777 suggests the absence of horizontal pleiotropy. Sensitivity analyses utilizing the leave‐one‐out method (Figure 4A) demonstrated no significant impact of individual SNPs on the outcomes. Consequently, it can be inferred that MUFAs do not exert a causal influence on the development of lung adenocarcinoma.

**TABLE 2:** Results of Mendelian randomization analysis for the three lung cancer pathological types.

A. Results of Mendelian randomization analysis for lung adenocarcinoma						
	Methods	Number of SNPs	Beta	*SE*	*P*‐value	OR (95% CI)
Results	MR Egger	51	0.03883	0.08154	0.636	1.040 (0.886 ~ 1.220)
	Weighted median	51	0.07092	0.07287	0.330	1.073 (0.931 ~ 1.238)
	Inverse variance weighted	51	0.05711	0.0499	0.252	1.059 (0.960 ~ 1.168)
	Weighted mode	51	0.05102	0.08023	0.528	1.052 (0.899 ~ 1.232)

B. Results of Mendelian randomization analysis for lung squamous cell carcinoma						
	Methods	Number of SNPs	Beta	*SE*	*P*‐value	OR (95%CI)
Results	MR Egger	54	−0.1014	0.152	0.508	0.904 (0.671 ~ 1.217)
	Weighted median	54	−0.03265	0.1061	0.968	1.073 (0.786 ~ 1.192)
	Inverse variance weighted	54	−0.1238	0.08563	0.148	0.884 (0.747 ~ 1.045)
	Weighted mode	54	0.04943	0.1388	0.723	1.051 (0.800 ~ 1.379)

C. Results of Mendelian randomization analysis for small cell lung cancer.						
	Methods	Number of SNPs	Beta	*SE*	*P*‐value	OR (95%CI)
Results	MR Egger	51	−0.06194	0.1838	0.738	0.940 (0.656 ~ 1.348)
	Weighted median	51	−0.2017	0.1489	0.176	0.817 (0.610 ~ 1.094)
	Inverse variance weighted	51	−0.06657	0.1123	0.554	0.936 (0.751 ~ 1.166)
	Weighted mode	51	−0.1316	0.1536	0.396	0.877 (0.649 ~ 1.185)

Abbreviations: Beta, beta coefficient; MR, Mendelian randomization; OR, odds ratio; SE, standard error; SNP, single nucleotide polymorphism.

### 3.2 Causal Relationship Between MUFAs and Lung Squamous Cell Carcinoma

Instrumental variables: According to the principle of “Screening of instrumental Variables” in this paper, 54 SNPs were finally included for subsequent Mendelian randomization analysis.

The MR analysis and sensitivity analysis employed the Cochran's Q test, which resulted in a *p* value of 0.00005558. The inverse variance weighting method with a random effects model was chosen, yielding a *p* value of 0.148. Furthermore, the MR‐Egger regression, weighted median, and weighted pattern methods all provided results consistent with the inverse variance weighting method, with all *p* values exceeding 0.05. The result of the IVW analysis was OR = 0.884, 95% CI (0.747 ~ 1.045) (Figure 2B, Figure 3B, Table 2B). The MR‐Egger regression intercept *p* = 0.858, indicating that there was no horizontal pleiotropy. Sensitivity analyses with the leave‐one‐out method (Figure 4B) showed no significant effect of individual SNPs on the outcomes. Thus, there was no causal effect of MUFAs on lung squamous cell carcinoma. Sensitivity analyses utilizing the leave‐one‐out method (Figure 4B) revealed that individual SNPs did not have a statistically significant impact on the outcomes, indicating the absence of a causal relationship between MUFAs and lung squamous cell carcinoma.

### 3.3 Causal Relationship Between MUFAs and Small Cell Lung Cancer

Instrumental variables: According to the principle of “Screening of instrumental Variables” in this paper, 51 SNPs were finally included for subsequent Mendelian randomization analysis.

The MR analysis included sensitivity analysis using the Cochran *Q* test, which yielded a calculated *p* value of 0.004721. The inverse variance weighting method with a random effect model was selected, resulting in a *p* value of 0.554. The MR‐Egger regression method, weighted median method, and weighted pattern method all showed consistency with the inverse variance weighting method, with all *p* values exceeding 0.05. The IVW analysis revealed an odds ratio of 0.936 with a 95% confidence interval of 0.751 to 1.166 (Figure 2C, Figure 3C, Table 2C). The MR‐Egger regression intercept yielded a *p* value of 0.975, indicating the absence of horizontal pleiotropy. Sensitivity analyses utilizing the leave‐one‐out method (Figure 4C) revealed that individual SNPs did not have a statistically significant impact on the outcomes, indicating that MUFAs do not exhibit a causal relationship with small‐cell lung cancer.

## 4 Discussion

This study conducted an analysis of the causal relationship between MUFAs and lung cancer across three distinct pathological subtypes of lung cancer utilizing a two‐sample Mendelian randomization approach. The results of the MR analysis indicated that there was no evidence of a direct causal association between exposure to MUFAs and the development of lung cancer.

The findings of this study suggest that the consideration of MUFAs exposure may have an indirect impact on the outcomes of lung cancer through various mechanisms [13]. Numerous studies have demonstrated the significant role of fatty acids in cancer development, as well as their effects on tumors, cardiovascular diseases, and inflammation, garnering considerable attention. MUFAs, including oleic acid, are essential components of dietary fat intake and cannot be synthesized by the human body, primarily sourced from certain vegetable oils as dietary supplements.

This is the first study to examine the relationship between monounsaturated fatty acids and lung cancer (especially of multiple lung cancer pathotypes) using Mendelian randomization. In this study, the direct causal relationship between fatty acids and lung cancer was not established, which aligns with the conclusions of several related studies. Among the existing studies on the relationship between fatty acids and lung cancer, PUFAs account for the majority of studies. For instance, Zhang et al. conducted a meta‐analysis that found no significant link between high PUFA consumption and lung cancer risk (RR 0.91, 95% CI 0.78–1.06) [14]. Moreover, specific fatty acids can have varying effects across different populations. Luu et al. suggested that EPA intake might be associated with a higher lung cancer risk in female non‐smokers [15], indicating that the influence of various fatty acids can differ among demographic groups. Additionally, Kojima et al. reported that high levels of omega‐3 PUFAs were inversely related to colorectal cancer risk, supporting potential biological mechanisms by which fatty acids may impact cancer development [16]. Although MUFAs do not seem to directly affect lung cancer risk, their balanced consumption in the diet remains essential. Pouchieu et al. highlighted that antioxidants could influence the carcinogenic effects of different fatty acids, underscoring the importance of interactions among dietary components [17]. Thus, future research should investigate the roles of specific fatty acids in various cancers and consider their interactions with other dietary elements, as recommended by Chajès et al [18]. In summary, these findings emphasize the need for a nuanced understanding of dietary fatty acids in cancer biology, guiding future dietary recommendations and cancer prevention strategies based on a more robust evidence base.

SCD1 plays a significant role in the conversion of saturated fatty acids to monounsaturated fatty acids, which is intricately linked to the levels of monounsaturated fatty acids. The expression of SCD1 is strongly associated with the unfavorable prognosis of lung adenocarcinoma, indicating that SCD1 may facilitate the synthesis of monounsaturated fatty acids and contribute to the poor prognosis of this cancer type [19]. In specific circumstances, the introduction of exogenous monounsaturated fatty acids could mitigate the deficiencies resulting from SCD1 depletion [20]. Moreover, polyunsaturated fatty acids play a crucial role in the process of oxidation and iron‐mediated cell death, particularly in the conversion of saturated fatty acids to monounsaturated fatty acids by SCD1 [21]. This conversion may indirectly impact the development of lung cancer by influencing lung adenocarcinoma tolerance. Additionally, STK11 (Serine/threonine kinase 11) mutation can enhance the synthesis of monounsaturated fatty acids to inhibit ferroptosis in LUAD cells [22]. OA has been shown to counteract the cytotoxic effects of lung cancer–targeting drugs in cell lines with activating epidermal growth factor receptor (EGFR) mutations [20]; meanwhile, OA can reduce syndecan 4 expression and facilitate ferroptosis in lung cancer cells via the glutathione peroxidase 4 (GPX4)/long‐chain acyl‐CoA synthetase (ACSL4) pathway, offering a strong rationale for employing ferroptosis‐targeting drugs in lung cancer therapy [23]. In a separate study, the overexpression of EGFR in lung cancer cell membranes has been shown to stabilize the SCD1 protein, leading to an increase in intracellular MUFAs and promoting lung cancer growth [24]. Additionally, MUFAs have been found to impact the expression of programmed cell death ligand 1 (PD‐L1), a crucial immune checkpoint molecule in lung cancer cells and immune cells [25]. The regulation of immune response by MUFAs may have implications for the prognosis of lung cancer. Ultimately, MUFAs may play a role in the pathogenesis and advancement of lung cancer via an indirect mechanism, resulting in a negative clinical outcome, in accordance with the findings of the current investigation.

This study demonstrates strengths in its utilization of genetic analysis to mitigate confounding factors, such as environmental, researcher, and social influences, as well as to eliminate the impact of reverse causality. Additionally, the use of public databases with large sample sizes for subgroup analysis ensures consistency in analysis and representative data, thereby reducing deviations stemming from population heterogeneity and pathological variations. Furthermore, various statistical methods, including the inverse variance weighting method, MR‐Egger regression method, weighted median method, and weighted model of comprehensive analysis, are employed to enhance the comprehensiveness and reliability of the results. The study is constrained by several limitations, including the potential introduction of confounding factors due to ineffective exclusion of weak instrumental variables in the screening process, a lack of data on the relationship between MUFAs and lung cancer in non‐European populations, and the inability to conduct more detailed grouping analysis of variables such as gender, age, and smoking status due to data limitations.

## 5 Conclusions

Collectively, the findings from the Mendelian randomization analysis indicate that there is no direct causal association between MUFAs and lung cancer. Nevertheless, it is possible that MUFAs may be influenced by iron levels or modulate immune responses, thereby indirectly impacting the progression of lung cancer. Further validation of these results is warranted through additional clinical and fundamental investigations.

## Author Contributions

Shaofeng Zhang and Jia Jiang made substantial contributions to the conception of the work and the acquisition and analysis of data; Xiping Wu and Xiang Liu drafted the work or revised it critically for important intellectual content; Xiang Liu and Qiang Xiao approved the version to be published; Wei Lei, Siqin Chen, Yaling Zeng, and Jiayi Liu contributed significantly to the analysis and manuscript preparation.

## Ethics Statement

The datasets were obtained from the GWAS database and all data were under ethics approval before recorded in the database.

## Conflicts of Interest

The authors declare no conflicts of interest.

## Data Source

MUFAs and lung cancer data were obtained from the GWAS database (https://gwas.mrcieu.ac.uk/), utilizing data sourced from a publicly available repository of peer‐reviewed research papers. The original ethical approval for this study has been obtained, rendering an additional ethical review unnecessary. Detailed information regarding the specific data can be found in Table 1. The MUFAs data was analyzed from a sample size exceeding 110 000 individuals. Conversely, the data on lung cancer was gathered from genotyping of 14 803 European patients with lung cancer and 12 262 controls, as well as from GWAS of 29 266 patients and 56 450 controls, categorized according to three distinct pathological types of lung cancer.

## Data Availability

In this study, publicly available datasets were utilized, and the data can be accessed at the following link: https://gwas.mrcieu.ac.uk/.
